# Risk of sensitization and allergy in Ragweed workers – a pilot study

**DOI:** 10.1186/1710-1492-10-42

**Published:** 2014-08-08

**Authors:** Oliver Brandt, Torsten Zuberbier, Karl-Christian Bergmann

**Affiliations:** 1 Department of Dermatology and Allergy, Charité-Universitätsmedizin Berlin, Charitéplatz 1, 10117 Berlin, Germany

**Keywords:** Ragweed, Sensitization, Pollen allergy, Allergic rhinoconjunctivitis, Allergic asthma, Occupational risk

## Abstract

**Background:**

Due to its high allergenic potential *Ambrosia artemisiifolia* has become a health threat in many European countries during the last few decades. Hence, several cities and communities initiated ragweed eradication campaigns. In Berlin, Germany, so-called Ambrosia scouts are being assigned the task of finding and eliminating this weed.

We sought to evaluate the potential risk of sensitization and allergy in these individuals.

**Findings:**

In order to assess the risk of sensitization and allergy, we followed-up 20 Ambrosia scouts by skin-prick test with inhalant allergens, immunoserological and pulmonary function tests. Additionally, medical conditions were evaluated by a questionnaire especially designed for this study.

Despite close contact to ragweed over a median duration of 13.8 months, none of the participants became sensitized or allergic to ragweed. One individual developed a clinical non-relevant sensitization towards the taxiconomically-related plant mugwort. A decline in relative FEV1 was most probably due to heavy smoking.

**Conclusions:**

Our surprising findings suggest that intensive contact and exposure to high ragweed pollen concentrations do not necessarily result in sensitization and/or allergy, meaning that the allergenic potential of this weed might be lower than hitherto expected. However, it is also conceivable that continuous exposure to high allergen levels induced tolerance in the ragweed workers.

Due to the relatively small number of subjects studied, our results might be biased and therefore investigations on larger study groups are needed.

## Background

During the last few decades common ambrosia (*Ambrosia artemisiifolia*) has become a health threat in many European countries [1]. Due to the huge amount of pollen produced, combined with their high allergenic potential, there is reason to fear that the spread of this plant could lead to an increase in the number of sensitizations and, subsequently, allergies to *A. artemisiifolia*[2].

Hence, several cities and communities in Europe decided to fight the invasion by initiating a ragweed eradication campaign [1,3]. In 2008 Berlin launched an initiative aiming to combat the spread of ambrosia plants within the metropolitan area. So-called “Ambrosia-scouts” were also assigned the ethically questionable task of finding and eliminating this vegetation. As these scouts, merely protected by disposable gloves and surgical masks, came in close contact with the plants even during the entire flowering season, we were interested in whether these subjects would become more sensitized to or even allergic towards ragweed and/or its taxonomically related weed mugwort.

## Methods

From November 2008 until June 2012 a follow-up study among ragweed workers in Berlin, Germany, was performed. Forty-five subjects (median age 44 years, 33% females) were recruited by an open invitation to participate in the study, monitoring their state of health immediately prior to or shortly after taking up work as Ambrosia Scouts. Data regarding age, gender, smoking habits, atopic symptoms, and chronic lung diseases (allergic or non-allergic asthma, chronic obstructive pulmonary disease) were collected via a questionnaire especially developed for this study. Participants were physically examined, skin-prick tests (SPT) were performed (ALK Abello, Wedel, Germany) [4] and levels of total and specific-IgE (sIgE) were determined (Phadia AB, Uppsala, Sweden) in order to gather sensitizations towards common inhalant allergens [5]. Additionally, pulmonary function parameters were assessed, as described previously [4-6].

All subjects consented and the study was approved by the Ethical Board at Charité University Medicine, Berlin, Germany.

## Findings

Of the forty-five individuals initially examined, 20 (44.4%) responded to our invitation and accepted to participate in the follow-up clinical evaluation (Table 1). Participants were employed as ragweed workers for a median average duration of 13.8 months (range 3-33) and had removed up to more than 1,000 plants. Four had a history of allergic rhinitis and/or allergic asthma, 3 of which with known sensitizations towards birch and/or grass pollen. No other chronic diseases were reported. Fourteen of the 20 participants (70%) were smokers.

**Table 1:** Demographic data and characteristics of the study group examined before commencement and at the end of their work as ragweed workers

	**Initial examination**	**Final examination**
**Number of subjects**	**20**	**20**
Sex (♀/♂)	06/14/14	06/14/14
Age (yrs)^1^	45.5	48.5
range (min-max)	29-59	32-61
Smokers	14 (70%)	16 (80%)
Allergic rhinits	3/20 (15%)	3/20 (15%)
Allergic asthma	0/20 (0%)	0/20 (0%)
Atopic dermatitis	0/20 (0%)	0/20 (0%)

^1^median values.

In the initial examination 6/20 (30.0%) participants showed positive SPT-results. Respectively 3/20 (15.5%) of them were sensitized towards birch and mugwort allergens, of which one mugwort allergic subject additionally exhibited a sensitization towards ragweed (Table 2).

**Table 2:** Diagnostic features and laboratory data of ragweed workers

	**Initial examination**	**Final examination**
**Number of subjects**	**20**	**20**
**Skin-prick tests**		
Hazel	2/20 (10.0%)	1/20 (5.0%)
Alder	2/20 (10.0%)	1/20 (5.0%)
Birch	3/20 (15.0%)	3/20 (15.0%)
Grass mixture	2/20 (10.0%)	3/20 (15.0%)
Mugwort	3/20 (15.0%)	2/20 (10.0%)
Ragweed	1/20 (5.0%)	1/20 (5.0%)
Derm. pteronys	0/20 (0.0%)	1/20 (5.0%)
mono-/polysensitized^b^	4/2	4/2
**Serum IgE**		
Total IgE kU/l^a^	44.1	41.5
range	1.9-1,146.0	2.4-1,362.0
Total IgE >100 kU/l	3/20 (15.0%)	2/20 (10.0%)
*Specific IgE (≥0.35 kU/l)*		
Birch t3	2/20 (10.0%)	2/20 (10.0%)
Timothy grass g6	2/20 (10.0%)	2/20 (10.0%)
Mugwort w1	1/20 (5.0%)	1/20 (5.0%)
Ragweed w6	0/20 (0.0%)	0/20 (0.0%)
Derm. pteronyssinus d1	1/20 (5.0%)	1/20 (5.0%)
mono-/polysensitized^b^	3/1	3/1
**Pulmonary-function-tests**		
*FVC % (FVC, L)*		
All^a^	98.0 (4.27)	106.5 (4.35)
Range	78-125 (2.56-5.95)	75-142 (2.52-6.19)
*FEV1% (FEV1, L)*		
All^a^	102.5 (3.22)	93.0 (3.25)
Range	77-115 (1.84-4.73)	76-127 (1.77-4.72)
*FEV1/FVC (%)*		
All^a^	81	76
Range	71-96	62-85

^a^median values.

^b^polysensitized was defined as more than one sensitization in a subject.

*FVC*, Functional vital capacity (L).

FEV1, Forced exspiratory volume in 1 sec.

FEV1/FVC, Relative forced exspiratory volume in 1 sec.

L, Liter.

At the follow-up visit 6/20 (30.0%) exhibited sensitizations towards the investigated allergens. Sensitizations to birch and grass pollen were the most common, while sensitizations to ragweed and mugwort were only detected in one, respectively two of the cases, who were already diagnosed as being sensitized in the initial examination.

Median total IgE values were 44.1 kU/l at the initial and 41.5 kU/l at the final visit. Values higher than 100 kU/l were detectable in 3 (15%) individuals in the initial visit, two of which additionally exhibited allergen-sIgE concentrations above 0.35 kU/l, and of these in turn one participant was sensitized towards mugwort. Since the initial visit one individual quitted smoking.

At the follow-up visit 2/20 (10%) exhibited total IgE levels above 100 kU/l and also sIgE antibody concentrations higher than 0.35 kU/l for one (timothy grass) and two (birch, timothy grass) antigens, respectively. One of the ragweed workers exhibited allergen-sIgE antibodies directed against mugwort (0.59 kU/l) in the final, but not at the initial examination. The participant in whom a sensitization towards mugwort was detected in the initial examination (sIgE 0.36 kU/l), no significant allergen-sIgE concentrations were found at the final visit. None of the individuals showed allergen-sIgE antibodies reactive with ragweed extracts neither before commencement nor after completion of work.

Investigation of pulmonary function parameters revealed a significant decline in relative FEV1 from 81 to 76% (p = 0.0065), while FVC and FEV1 did not exhibit relevant changes. None of the workers with pre-existing asthma, however, developed exacerbations or complained of dyspnea at any time during their employment.

## Discussion

Despite intensive and frequent contact with ragweed - even during pollination - none of the 20 Ambrosia scouts examined became sensitized or even allergic to ragweed over a median period of 13.8 months. These findings are surprising as a single ragweed plant produces millions of pollen grains that are highly allergenic [7,8]. Tosi et al. [7] investigated the prevalence of sensitizations and allergies towards ragweed in the Milan region (northern Italy) and found steadily increasing rates. Moreover, unlike with other aeroallergens, exposure to ragweed antigens frequently resulted in sensitizations and allergies, respectively, even in individuals over the age of fifty. This is noteworthy as eight of the twenty ragweed workers investigated were older than 50 years upon commencement of this work. It is also remarkable that 14/20 subjects are males, as according to Rueff et al. [8] male gender is a strong predictor for ragweed sensitization. Furthermore, one male ragweed worker aged 59 who was already sensitized at the initial visit, didn’t develop allergic symptoms during employment. We can only speculate about these contradictory findings.

Firstly, there are time lags between the exposure towards an allergen and the development of a sensitization as well as between the sensitization and the progression towards allergy [7]. Although the periods are variable and their exact latencies unknown, it is conceivable that the time of exposure was not sufficient to become sensitized. Moreover, it is quite possible that allergenicity of ragweed pollen is overestimated. Ackermann-Liebrich et al. [9] compared the sensitization rates between 1991 and 2002 in different regions of Switzerland and found that even in areas with high ragweed pollen loads no significant increases of sensitization rates could be detected. They therefore concluded that Ambrosia plants “do not yet represent an important public health issue” in Switzerland.

Investigation of clinical variables with ragweed sensitization revealed that the number of existing sensitizations is the most critical determinant, meaning that polysensitized individuals are more prone to become sensitized to a new antigen than non- or monosensitized subjects [8]. As the majority of the ragweed workers were not sensitized to any allergen and only six – three of which were polysensitized – exhibited sensitizations, low susceptibility might be another reason for our unforeseen results. In other words: a higher number of atopic individuals could have resulted in higher sensitization rates.

Secondly, in this study 70% of the individuals were smokers at the initial visit. This is of importance as smoking has been demonstrated to have a significant impact on the immune system. In a population-based cohort study Hancox et al. [10] found cigarette smoking in teenage and early adult life to be associated with a lower risk of allergic sensitization in those with a family history of atopy.

As the ragweed workers were instructed to wear face masks while executing their mission it is also possible that this security measure prevented them from becoming sensitized and allergic, respectively. In a study from Japan the protective effect of such masks on nasal cavity pollen exposure was investigated [11]. While there was a significant reduction in the number of pollen particles in the nasal cavity of subjects with face masks, this result correlated with wind speed. The authors therefore concluded that their finding might only be true for certain wind velocities. Furthermore, due to the high pollen grains release rate of ragweed plants one can assume a surpassing pollen exposure of the ragweed workers despite the wearing of face masks.

Fourthly, it is also conceivable that the workers became tolerant to ragweed allergens. A number of studies have demonstrated that continuous exposure to high concentrations of an allergen modulates the immune system, eventually resulting in tolerance induction. Investigations in young children exposed to cat allergens showed that medium levels of Fel d1 led to sensitization while low concentrations did not induce an IgE response and high allergen levels resulted in tolerance induction [12,13]. Riedl et al. [14] exposed atopic volunteers nasally to different doses of keyhole limpet hemocyanin (KLH), an immunostimulatory protein and neoantigen widely employed in vaccines, and found antigen-specific IgG and IgG4 antibodies with the highest levels in the high-dose group indicating tolerance induction.

One may hypothesize that geographical factors or location of plants (e.g. inner city) might have an impact on the allergenicity of its pollen. It has been shown, however, that although ragweed plants within a population exhibit a high genetic diversity, only small differences across populations of various regions exist [15,16]. Furthermore, Ghiani et al. [17] recently demonstrated that ragweed pollen sampled along high traffic roads exhibit a significantly higher allergenicity than those from vegetated areas, presumably because of pollutants interacting with the pollen’s cell wall structure thereby enhancing the release of antigens. Accordingly, one might expect that the pollen of ragweed plants in inner cities possess a high allergenic potential.

Except for relative FEV1, lung function parameters did not change significantly. As neither sensitizations and/or allergies to ragweed nor respiratory symptoms occurred, the significant decline seen in relative FEV1 was most likely attributable to continuing nicotine abuse, as heavy smoking can result in declined lung function parameters within a short period of time [18].

Lastly, we are aware that our findings might be biased by the relatively small sample size and the fact that the individuals studied originate from precarious social conditions. Thus, investigations of larger study groups with individuals from diverse social backgrounds frequently exposed to high amounts of ragweed pollen are needed.

## Conclusion

Altogether, the data of our pilot study suggest that frequent and intensive exposure towards ragweed pollen not necessarily results in sensitization and allergy. However, these findings may be biased by the relatively small sample size studied, the low number of atopic individuals, and the social background of the subjects. Therefore, confirmation of our findings in larger subject cohorts is mandatory.

## Abbreviations

COPD: Chronic obstructive pulmonary disease; FEV1: Forced exspiratory volume; FVC: Forced vital capacity; KLH: Keyhole limpet hemocyanin; sIgE: Specific immunoglobulin E; SPT: Skin prick test.

## Competing interest

The authors declare that they have no competing interest.

## Authors’ contributions

Acquisition, analyses, and interpretation of the data were carried out by OB and KCB. The manuscript was written by OB and edited by KCB and TZ. All authors read and approved the final manuscript.
